# Age-growth relationships, temperature sensitivity and palaeoclimate-archive potential of the threatened Altiplano cactus *Echinopsis atacamensis*

**DOI:** 10.1093/conphys/coaa123

**Published:** 2021-01-27

**Authors:** N B English, D L Dettman, Q Hua, J M Mendoza, D Muir, K R Hultine, D G Williams

**Affiliations:** 1 School of Health, Medical and Applied Science, Central Queensland University, 538 Flinders St West, Townsville, QLD 4810, Australia; 2 Department of Geosciences, University of Arizona, Tucson, AZ 85721, USA; 3 Estuary Research Center, Shimane University, Matsue, 690-8504, Japan; 4 Australian Nuclear Science and Technology Organisation, Lucas Heights, NSW 2234, Australia; 5 Herbario del Oriente Boliviano (USZ), Museo de Historia Natural Noel Kempff Mercado, Av. Irala 565, Casilla 2489, Santa Cruz, Bolivia; 6 Murdoch University, Perth, WA 6009, Australia; 7 Department of Research, Conservation and Collections, Desert Botanical Garden, Phoenix, AZ 85008, USA; 8 Department of Botany, University of Wyoming, Laramie, WY 82071, USA

**Keywords:** Acanthochronology, Altiplano, cactus, climate, stable isotopes

## Abstract

The tall (>4 m), charismatic and threatened columnar cacti, pasacana [*Echinopsis atacamensis* (Vaupel) Friedrich & G.D. Rowley)], grows on the Bolivian Altiplano and provides environmental and economic value to these extremely cold, arid and high-elevation (~4000 m) ecosystems. Yet very little is known about their growth rates, ages, demography and climate sensitivity. Using radiocarbon in spine dating time series, we quantitatively estimate the growth rate (5.8 and 8.3 cm yr^−1^) and age of these cacti (up to 430 years). These data and our field measurements yield a survivorship curve that suggests precipitation on the Altiplano is important for this species’ recruitment. Our results also reveal a relationship between night-time temperatures on the Altiplano and the variation in oxygen isotope values in spines (δ^18^O). The annual δ^18^O minimums from 58 years of in-series spine tissue from pasacana on the Altiplano provides at least decadal proxy records of temperature (*r* = 0.58; *P* < 0.0001), and evidence suggests that there are longer records connecting modern Altiplano temperatures to sea-surface temperatures (SSTs) in the Atlantic Ocean. While the role of Atlantic SSTs on the South American Summer Monsoon (SASM) and precipitation on the Bolivian Altiplano is well described, the impact of SSTs on Altiplano temperatures is disputed. Understanding the modern impact of SSTs on temperature on the Altiplano is important to both understand the impact of future climate change on pasacana cactus and to understand past climate changes on the Altiplano. This is the best quantitative evidence to date of one of the oldest known cactus in the world, although there are likely many older cacti on the Altiplano, or elsewhere, that have not yet been sampled. Together with growth, isotope and age data, this information should lead to better management and conservation outcomes for this threatened species and the Altiplano ecosystem.

## Teaser

Isotopes in spines of the giant pasacana cactus on the Bolivian Altiplano provide important ecophysiological information for management of this foundation species and record climate information related to Altiplano temperatures and cactus demography.

## Introduction

**Figure 1:**
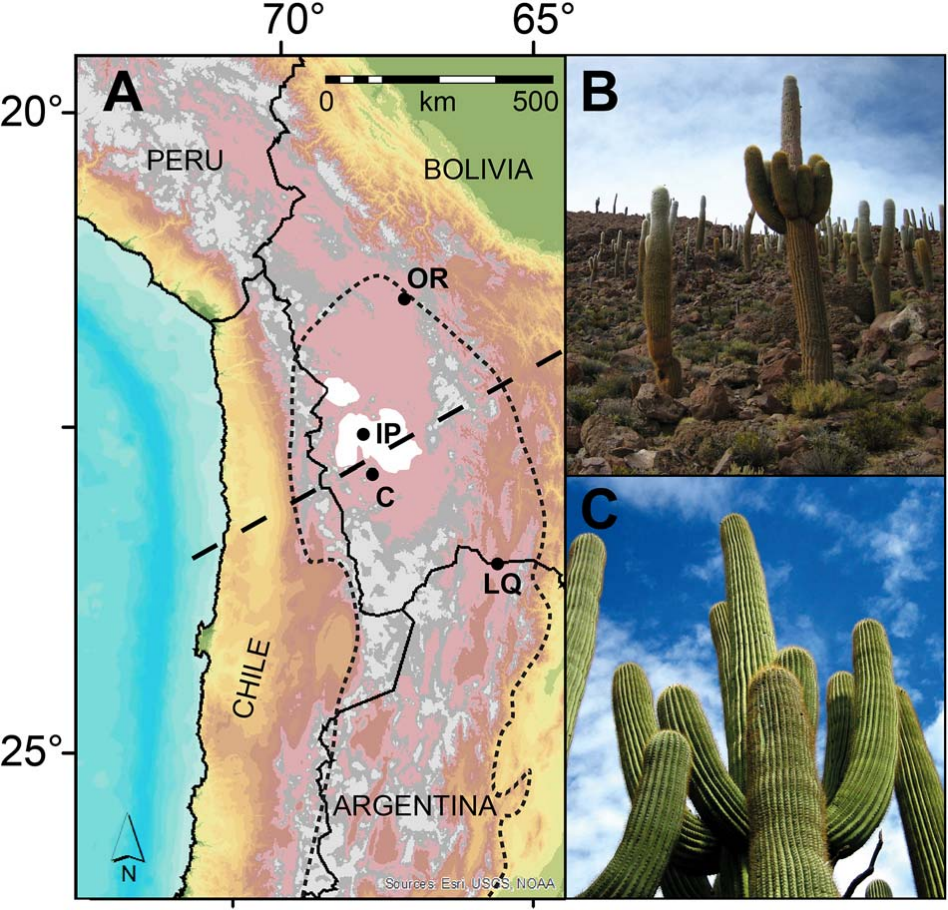
(A) Digital elevation model of the Altiplano (grey is >4000 m asl) and the location of the Salar de Uyuni and Coipasa (southern and northern white areas, respectively), Isla del Pescado (IP), Oruro (OR), Colcha (C) and La Quiaca (LQ). The estimated range of pasacana cactus is indicated by the short-dashed line. The rough boundary between ENSO and Gran Chaco climate regimes is indicated by the straight-dashed line. (B) *Echinopsis atacamensis* (pasacana) on Isla del Pescado, Bolivia. (C) *Carnegiea gigantea* (saguaro) in Tucson, USA. DEM was created by Dr Michael Hewson using Esri Inc. ArcGIS 10.6 and US Geological Survey’s Centre for Earth Resources Observation and Science topographic data (*Data available from the U.S. Geological Survey)*.

On the southern Altiplano, the long-lived columnar cactus pasacana [*Echinopsis atacamensis* var. *pasacana*, (Phil.) H. Friedrich & G.D.Rowley] rely on strongly seasonal precipitation over the Altiplano for their growth, with the majority of precipitation (mean annual precipitation, >200 mm yr^−1^) occurring during the austral summer (DJF) and from the east (Morales *et al.*, 2012). Populations of pasacana found on the Southern Altiplano (Fig. 1b) are considered threatened, or near threatened, due to the limited suitability of high altitude (between 2000 and 4000 m asl), cold and dry habitat (mean annual temperature between −0.6° and 16.4°C; 200 mm yr^−1^ precipitation) and their value as the only readily available local timber (Yetman, 2008). Pasacana share an anatomy, physiology and ecological function similar to the North American Saguaro cactus (*Carnegiea gigantea*, Fig. 1c). Specifically, both species are slow-growing, long-lived and can store massive amounts of water in their succulent tissues. Both species produce a single stem at the base and do not produce secondary arms for decades after emergence. The arms usually form 2 to 3 meters above ground level (Fig. 1b,c). Like saguaro, pasacana provide multiple ecologic and economic services (Gibson *et al.*, 1986; Nobel, 2002; Yetman, 2008) for the Altiplano in addition to being potential archives of climate information (English *et al.*, 2007; English *et al.*, 2010a; English *et al.*, 2010b; Hultine *et al.*, 2018).

**Figure 2:**
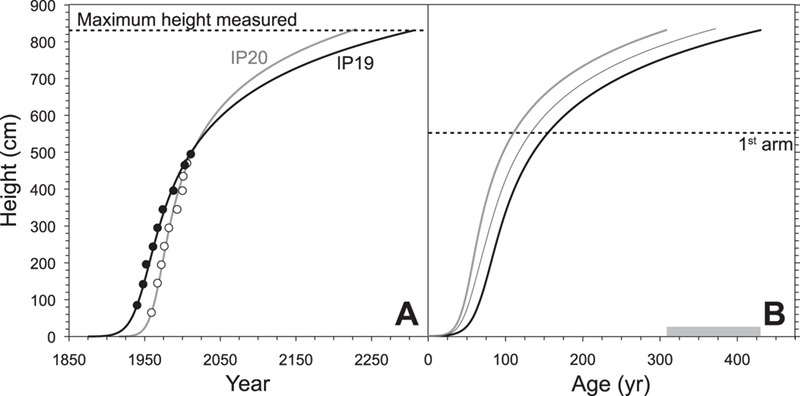
Height and ^14^C-age of spines from two pasacana cactus on the Isla del Pescado. (A) Solid circles (black line) and open circles (grey line) are spines and best fit growth models for IP19 and IP20, respectively. The maximum height (830 cm) of cactus measured at this site is denoted by the dashed line. (B) Growth model for IP19 (black), IP20 (grey) and their mean (thin). Grey box at bottom indicates the range of ages (308 to 430 years) at which IP19 and IP20 growth curves intersect 830 cm, the height of the tallest pasacana measured at this site. The median height (550 cm) of a cactus with one arm (first arm) is indicated by the dashed line.

Traditionally, measurements of cactus stem growth have taken place over years or decades (Drezner, 2003b; Drezner, 2005; Pierson *et al.*, 2013) using photographs or repeated measures, but the remote location of pasacana makes this work difficult and is one reason very little is known about their life history or physiology. English *et al.* (2007, 2010a, 2010b) showed that radiocarbon (^14^C) and δ^13^C in spines can be used to accurately determine the age and height of a cactus at any time from the 1950’s onwards, and advances in radiocarbon interpretation have increased the utility of this procedure (Hua, 2009)—a subdiscipline known as acanthochronology. These studies are aided by the growth habit of columnar cacti; spines emerge from the apex and mature in just a few weeks (English *et al.*, 2010a) where they remain in situ and in series as the apex continues to grow upward and produce more spines above (Gibson *et al.*, 1986). Measurements of cactus spine age along the side of a plant can be used to construct the growth history of individual plants and, using the age-height relationship of multiple plants in a population, allows a model of the population’s average growth rate to be developed. This growth model, combined with a large sampling of cactus heights in the same population, allows the age estimation of each cactus in the population and the development of a survivorship curve for the population. Peaks and troughs in recruitment revealed in the survivorship curve can be used to infer what variable (e.g. precipitation) might drive recruitment in this species.

The water balance in cactus is similar to that in lakes, where water storage in stems of cacti responds to changes in temperature, precipitation and vapor pressure deficit, that affects stem water recharge via roots and loss through transpiration (Hultine *et al.*, 2019). Their succulent stems swell or shrink during rainfall and drought, and the stem water isotope values shift predictably in response to these recharge and loss events (English *et al.*, 2010a). Like in other cacti, pasacana spines record the balance of water recharge and loss in the stem as they grow. The spines also record the stable isotope composition of the carbohydrates (δ^13^C and δ^18^O) used in their production, themselves a reflection of the plant’s photosynthetic processes and water balance (English *et al.*, 2007; English *et al.*, 2010a; English *et al.*, 2010b; Hultine *et al.*, 2019). Therefore, spines along a single vertical rib record a detailed and time-ordered sequence of isotopic variation over the life of the plant reflecting climate variability and physiology during growth. For long-lived cacti like pasacana, spines likely retain an archive of climate information and physiological responses throughout the life of the plant.

Similar to pasacana, precipitation and temperature have a large impact on the Altiplano’s water budget and the extent of the large ephemeral lake systems of the Salar de Uyuni and Salar de Coipasa (Lenters *et al.*, 1997; Garreaud *et al.*, 2009; Morales *et al.*, 2015; Satgé *et al.*, 2017). Many of these ephemeral lakes yield proxy records of millennial climate change, significantly longer timescales than a cactus (Placzek *et al.*, 2009). In the late Pleistocene, the salars appear to fill during North Atlantic sea-surface temperature (SST) cold events (*i.e* Heinrich events) and it is presumed that this is due to increases in precipitation associated with a southern displacement of the inter-tropical convergence zone (ITCZ), the equatorial atmospheric region associated with strong convection and precipitation. All else being equal, temperature has an exponential effect on evaporation rates for lakes and for cactus, whereas the effects of relative humidity (water vapor pressure) and precipitation on lake levels are linear (Gonfiantini, 1986).

Because there are so few data about any aspect of pasacana’s physiology or demography, the relative impact of these variables and a changing climate on their recruitment and survival in the future are unknown. This study addresses issues of pasacana demography, physiology and Altiplano climate by exploiting the in-sequence growth of spines and measurements of their radiogenic (^14^C) and stable (δ^13^C and δ^18^O) isotope compositions to reconstruct the water status and growth rates of two cactus (IP19 and IP20) from the Isla del Pescado in the Salar de Uyuni, Bolivia (see Materials and methods and Supplemental Data for details). We convert the growth rates into an estimated growth model that is used to create a survivorship curve of a small population of pasacana surveyed on Isla del Pescado to attribute the variations in spine isotopes to either temperature or moisture and to place δ^18^O minimum variations in temporal context.

## Materials and methods

### Field sampling

We measured the heights of 462 pasacana cactus over a roughly 100 m by 100 m plot (0.01 km^2^; 0.04 pasacana m^−2^) on Isla del Pescado, Salar de Uyuni, Bolivia (Fig. 1, 67.8090°W, 20.1410°S), and collected spine series from two ~5 m tall pasacana in August 2011 (IP 19 *n* = 224 spines for stable isotopes, *n* = 9 spines each from IP19 and IP20 for ^14^C measurements). Cactus heights were measured to the nearest 5 cm increment using telegraphing steel poles marked with tape (measurements were made by an uphill observer at eye level with the top of the plant). For ^14^C and stable isotope analyses, we clipped one spine from each areole along a single rib (1 areole per ~1.5 cm) on the south side of each cactus for the entire height of the cactus using methods described previously (English et al., 2007). The height above ground level for each spine sample was recorded with a cloth measuring tape attached to the cactus’ apex. Cacti were selected based on being single-stemmed, on a slope, as tall as we could reach the top of with our ladder safely, visually healthy and good representatives of cactus in the area. For ^14^C analyses we collected additional spines from the same areole every 50 cm. We placed sampled spines in coin envelopes on which the height above ground and the cactus’ identification was recorded. Ladder height, safety and permit limitations prevented us from collecting samples from >5 m above ground level.

### Radiocarbon data and growth modelling

Spine samples from 2 cacti IP19 and IP20 (Supplemental Data Table 1) were cleaned using the standard acid-alkali-acid method before being combusted and converted to graphite (Hua *et al.*, 2001) for accelerator mass spectrometry ^14^C analysis using the STAR facility at ANSTO (Fink *et al*., 2004). Radiocarbon values are reported as fraction of modern carbon (F^14^C) (Reimer *et al.*, 2004) after normalization to 95% of oxalic acid I (HOx-I) standard, and correction for backgrounds (both accelerator and chemistry) and isotope fractionation using measured δ^13^C. Calendar ages of spines from each cactus were modeled using the Bayesian simple sequence (SS) model (Bronk Ramsey, 2008) in OxCal (Bronk Ramsey, 2009) with the date of sampling (August 2011) as the top boundary, and the Southern Hemisphere Zone 1-2 bomb radiocarbon data (Hua *et al*., 2013) extended back in time by the SHCal13 calibration curve (Hogg *et al.*, 2013) (Supplemental Data Table 1). The SS model uses only constraints in the chronological ordering of dates (samples at higher heights are younger than those at lower heights) without any assumption on cactus’ growth rate. The agreement indices (A_model_) of the two models were good (62.9% for IP19 and 70.3% for IP20) and are higher than the accepted level of 60% (Bronk Ramsey, 2008). For columnar cactus, spines emerge and grow near the apex of the plant, so the height of a spine is a direct indicator of the height of the apex at the time the spine was grown (i.e. the spine’s ^14^C age).

We calculated growth per year (*g*) using the ^14^C ages and heights of spines (*h*). A stepwise regression (Matlab, R2011b, The Mathworks Inc.) with log(*g*) as the dependent variable and *height*, log(*h*)...log(*h*) (Drezner, 2003a) as independent variables did not yield any significant models at α = 0.05, unsurprising given the small number of data points. While not significant, a very liberal (α = 0.5) stepwise regression on log(*g*) yielded a model where log(*h*) and log(*h*) (Drezner, 2003a) have large effects on growth, as seen in saguaro growth models. The saguaro growth model does not apply to these cacti; however, we view its form as a generalized growth model for columnar cacti and so use the polynomial form as a starting point to develop the pasacana model (Drezner, 2003a):

(1) \begin{equation*} \log{(g)}_t=a+b+c \left[\log{(h)}_t\right]+d {\left[\log{(h)}_t\right]}^9 \end{equation*}

where growth (cm) in any year can be estimated for any height (cm), *b* is an adjustable dummy variable to account for variable growth rates among individuals or populations and *a*, *c* and *d* are constants representing the species’ growth model (i.e. the shape of the growth curve). By optimizing *a*, *c* and *d* until the estimated growth matched the measured growth between intervals (as determined by ^14^C) we derived an estimated growth model that best fit the cactus measured at this site and can be used to estimate past and future growth (Supplemental Data Table 2). We emphasize that (1) the saguaro growth model is based on two heights from hundreds of cacti while our estimated pasacana growth model is based on 9 and 10 heights from two individual cacti, respectively and (2) the estimated growth model for pasacana, while the same shape, is different than the growth model for saguaro, not just an adjustment of the saguaro growth model. To be conservative, the growth curve begins at 3 cm (3 cm tall cactus = 1 year old). It is likely that this has the effect of slightly underestimating the age of a cactus by one or two years, but reduces the error in this part of life and likely accounts for stem shortening due to growth compaction of the stem at the base. To evaluate whether peaks in our survivorship curve were real or noise, we used the Silverman test package (R-stats, package ‘silvermantest’, Schwaiger and Holzmann, 26 July 2013) on both non-transformed height data (*h*) and natural log transformed height data (Ln(*h*)) (Silverman, 1981; Hall *et al.*, 2001).

### Stable isotope data

For the isotope analyses, we used ~1–2 mm of spine tissue from the pointy end of the spine. The spines are difficult to split lengthwise, so the first millimeter was used for δ^13^C and the next millimeter was used for δ^18^O; in our experience this probably means the analyses are being derived from material produced a day or less apart (English *et al.*, 2010a). In previous studies, we found that extracting α-cellulose from spines was unnecessary when looking at stable isotope variation over time (English *et al.*, 2007).

Carbon (δ^13^C) and oxygen (δ^18^O) stable isotope values of spines from one cactus (IP19) were measured at the Cairns Analytical Unit (James Cook University, Australia) and the Environmental Isotope Laboratory at the University of Arizona (USA), respectively. For funding reasons, we were restricted in the number of δ^18^O samples we could run and so this series is shorter than the δ^13^C series. For δ^13^C (VPDB), we measured ~ 200 μg of dried spine tissue with a CHN elemental analyser (Cosech Analytical Technologies, CA). For δ^18^O (VSMOW), we measured ~ 125 μg of dried spine tissue with a Thermal Conversion/Elemental Analyzer (Thermo Electron Corp, Waltham, MA). Both peripherals were attached to a Delta-plus (Thermo Electron Corp, Waltham, MA) continuous flow isotope ratio mass spectrometer. Repeated measurement of our Sigma-Aldrich cellulose working-standards yielded an experimental precision for δ^13^C and δ^18^O of 0.2‰ and 0.15‰, respectively. For this work, we also analyzed a homogenized spine internal standard for δ^13^C throughout the analytical runs (−12.13‰, σ = 0.26‰, *n* = 3). We did not analyze duplicate samples or a spine internal standard for δ^18^O for this work. For δ^18^O, and because our samples are relatively positive for plant samples, we analyzed two working standards in each δ^18^O analytical run (Benzoic Acid, *n* = 12, Sigma-Aldrich cellulose *n* = 10) and one international standard (IAEA Benzoic 601, n = 4). The typical standard deviation (σ) of the Benzoic and Sigma-Aldrich cellulose working standards distributed throughout the analytical runs was 0.3‰ and 0.15‰, respectively, and ~1‰ for IAEA Benzoic 601.

To increase the robustness of our interpretations, we processed stable isotope data (Supplemental Data: Stable Isotope Data) in several ways for comparison to the climate datasets. These methods are laid out in great detail in English *et al.* (2010b). The best way to describe the treatment of our stable isotope data is in terms of unmodelled and age-modelled data. Unmodelled data are defined as stable isotope ratios of spines associated with a particular height along the stem (Fig. 5a). Before applying the age model, we removed age effects and other linear trends from both time series using a simple linear regression model. Linear trends, including age effects, were generally small across the time series (<0.5‰ for ^13^C, < 2‰ for ^18^O). Data associated with calendar years (i.e. age-modelled data) are defined as isotope data that has been age-modelled using only the ^14^C dates to redistribute and interpolate stable isotope ratios. The Suess effect (the reduction of the atmospheric δ^13^C values due to fossil fuel use since the industrial revolution) has been removed from the dated δ^13^C time-series. Finally, we extract annual minimum, maximum and mean values from the dated stable isotope time-series and perform several analyses of the annualized stable isotope data with annualized climate data (see below) using both dated stable isotope data and the same data that has been linearly detrended (in addition to removing the Suess effect). We removed any linear trends in both isotope and temperature records to remove the influence of increasing global temperatures on their correlation over the length of record.

We transformed (‘annualize’) raw dual isotope data from spines into ‘annual’ data (Supplementary Information: MATLAB Code) based on the ^14^C ages (Supplemental Data Table 1) for IP19 to interpolate and evenly redistribute spine isotope data (Supplementary Data: Stable Isotope Data) using the method described in detail previously by English *et al.* (2010b). We note that the spine δ^13^C values in some cactus plants, such as saguaros, record an annual growth cycle as the spines from one areole are progressively enriched in ^13^C relative to the previously produced areole from the same year (English et al., 2007; Hultine et al., 2018). This enrichment pattern likely reflects seasonal changes in climate conditions during the growth period. The annual cycle in δ^13^C values observed in saguaro spines have been used as an annual marker, but annual carbon isotope cycles are not obvious in these pasacana cacti, and so the age-modelled isotope data are only roughly assigned to specific years.

### Climate data and analyses

Local and continuous instrumental climate data from the Salar de Uyuni region of the Altiplano does not exist prior to 1980. In our comparison of spine isotope records to climate and SST we use the following five sources: (1) 0.5° gridded temperature and precipitation data from the Climate Research Unit (CRU) (Harris *et al.*, 2014); (2) instrumental temperature data recorded at La Quiaca, Bolivia (1956 to 2011, Claris ID 10001) from the Claris/LPB website (http://wp32.at.fcen.uba.ar/gridded) (Tencer *et al.*, 2011); (3) breakpoint adjusted monthly temperature data from Oruro 250 km to the northeast of Isla del Pescado (1943–2011, available from http://berkeleyearth.lbl.gov/stations/152744); (4) monthly precipitation records from Colcha on the southern edge of the salar 68 km from Isla del Pescado (1980–2001, personal comm. D. Christie) (Morales *et al.*, 2012); and (5) the 1° gridded Hadley Centre Sea Ice and Sea Surface Temperature data set (HadISST) (Rayner, 2003). La Quiaca is at 3640 masl and, even at 330 km from Isla del Pescado, is the closest station for which a decades long instrumental record of temperature is available (Garreaud *et al.*, 2009; Tencer *et al.*, 2011). In order to examine the impact of climate dataset choices on our interpretations, we use both a regridded observational dataset (CRU 3.21) (Harris *et al.*, 2014) and the instrumental temperature data from La Quiaca and Colcha. All the climate datasets are monthly, and so we used KNMI climate explorer to not only reduce each series to an annual mean of monthly values, we initially chose the year to be from July to June of each year to better represent southern hemisphere seasonality (i.e. the hydrological year). We also ran all analyses with both datasets (isotope and climate) linearly detrended. There are important differences in the relationship of the La Quiaca temperature record and SSTs depending on the choice of annualization (July–June or January–December). The near-annual nature of the isotope spine series mimics this uncertainty, and we believe this is reflected by the sharing of many spatial field correlations of the spine isotope series with both the July–June and January–December annualization of La Quiaca and SST (not shown). There is a great deal of information lost in these transformations, but we believe that this is conservative with respect to the alternative; transforming stable isotope data from one cactus into monthly values even though the years are best estimates and most oscillations in the unmodelled isotope data are represented by ~ 4–8 spines.

## Results

**Figure 3:**
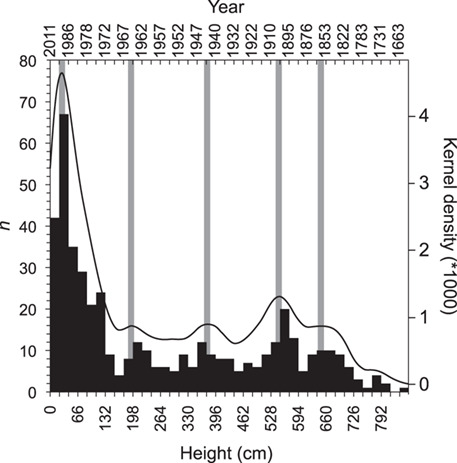
Survivorship curve of pasacana (*n* = 462) on Isla del Pescado. Height bins (bottom axis) are 22 cm and modeled pasacana ages (using the mean pasacana growth curve in Fig. 2) is shown on the top axis (non-linear). Black line shows kernel density of the height data and grey bars denote significant modal peaks (α = 0.06), although it is unclear if increased recruitment (peaks) or increased mortality (troughs) are their cause.

In this study, radiocarbon analyses of in-series and in-situ spines yield consistent increases in height with time in the last half of the century for both pasacana cacti measured (Fig. 2a, Supplemental Data Table 1). Growth rates for IP19 and IP20 were 5.8 and 8.3 cm yr^−1^ on average over ~ 70 and ~50 years, respectively. These rates are similar to saguaro growth rates in Tucson, AZ for the first ~ 80 years of cactus growth (7.3 to 11.5 cm yr^−1^; see Supplementary Information) (Drezner, 2005). However, the tallest pasacana of 462 plants measured was only 8.3 m tall, whereas saguaro cactus often exceed 12 m in height and can grow to >18 m tall. Using a dummy variable (*b*), we attempted to fit the saguaro growth model (Drezner, 2003a) to the spine F^14^C and height data from pasacana, however, the poor fit of the saguaro model to the pasacana data suggests that the growth model for pasacana is different (Supplemental Data Fig. 1). Therefore, we did not use the saguaro model and instead opted for a model that maintains the polynomial form (Fig. 2, Supplemental Data Table 2) and the slow-fast-slow growth of columnar cactus (Supplemental Data Fig. 2) (Yetman, 2008). Extrapolating from the pasacana model, we estimate the tallest cactus at this site (8.3 m tall) is between 308 to 430 years old (Fig. 2b). This new growth rate information allowed us to build a survivorship curve with peaks in survivorship near 1993, 1965, 1943, 1904 and 1862 (Fig. 3).

**Figure 4:**
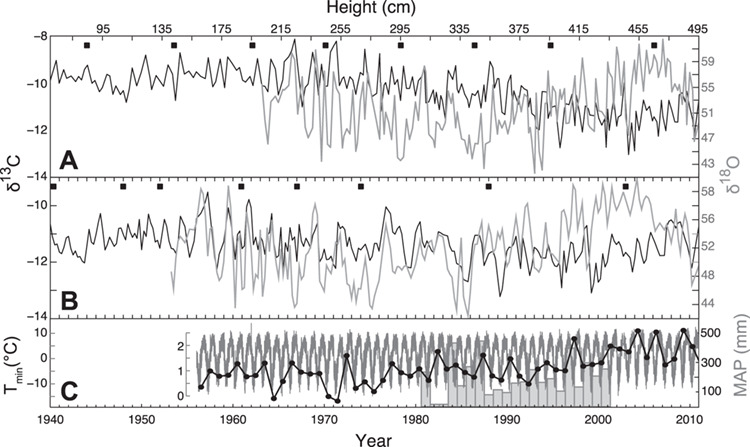
Stable isotope data from spines of IP19 vs climatic data from Colcha (precipitation) and La Quiaca (Temperature. (A) Spine heights and unmodelled stable isotope data. (B) Age-modelled (see Methods for more details) stable isotope data. For panels (A) and (B), closed squares are ^14^C dated spines, black and grey lines represent δ^13^C and δ^18^O, respectively, and the Suess Effect has been removed from δ^13^C data in panel B only. (C) Daily (dark gray line), mean annual monthly minimum temperatures (black line and circles) from La Quiaca and mean annual precipitation (bars) from Colcha^14^.

Dual measurements (δ^13^C and δ^18^O) of stable isotope variation in the spines of one cactus (IP19) span 58 years from 1953 to 2011 (Fig. 4, Methods), although the last year (2011) is incomplete and so not used in further analyses. Unmodelled carbon isotope ratios varied by ~ 5‰ (−13.0‰ to −8.1‰) over the period of measured record from 1940 to 2011. Unlike saguaro, the pasacana cactus did not yield a time-series with annual resolution (i.e. there were fewer δ^13^C oscillations visible than ^14^C results suggest should be visible) (English *et al.*, 2010a). We interpolated the age of spines between ^14^C samples (see Methods), and so this record is best described as near-annually dated. Unmodelled oxygen isotope ratios show extreme variation, >20‰ over the period of measured record (from ~1953 to 2011, 41.6‰ to 62.5‰). This is unlikely due to variation in the δ^18^O of rainfall, as this is thought to vary by only ~ 6‰ in the north central Andes and has no significant relationship to the amount of precipitation or temperature (Insel *et al.*, 2013). In fact, there is no significant correlation of annual maximum, mean or minimum δ^18^O to July–June precipitation at Colcha or with the regridded July–June precipitation (CRU 3.21) over the southern Altiplano region. The extreme variation in spine δ^18^O values must arise from varying degrees of plant water evaporation, recharge and residence time (offset by a + 27‰ fractionation between water and organic matter oxygen) (McCarroll *et al.*, 2004).

**Figure 5:**
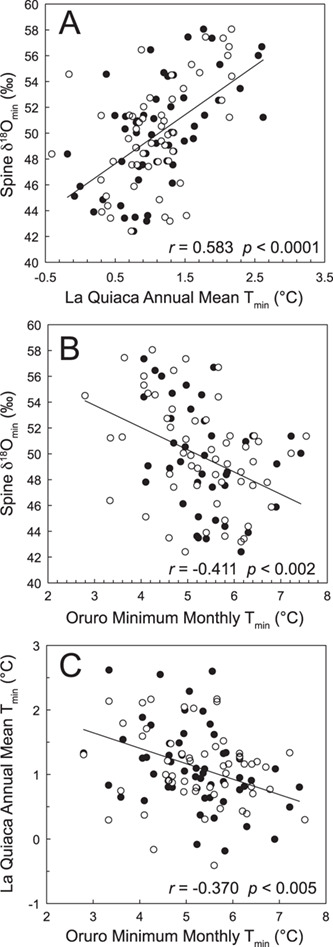
Relationship of cactus annual spine δ^18^O minimums (age modeled) and July–June (closed circle) and January–December (open circle) annual means of monthly minimum temperatures at La Quiaca (A) and minimum annual mean temperature at Oruro (B) between 1956 and 2010. Regression lines are for temperatures using July–June annualization and are indistinguishable from the trend line for temperatures using January–December annualization in both figures. Minimum temperatures (July–June) at La Quiaca and Oruro are negatively correlated (C). Regression lines and statistics are for simple linear regressions. Some circles lay directly on top of each other and are not both visible (e.g. in B, at 2.8°C and ~54‰ there is an open and closed circle).

**Figure 6:**
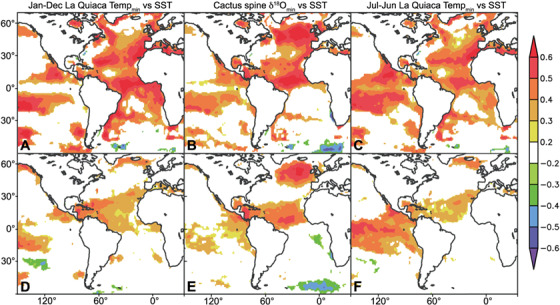
Spatial correlation field of annual means (January–December) of monthly minimum temperatures at La Quiaca (A, D), annual minimum δ^18^O of spine tissue (B, E) between 1953 and 2010, and annual means (July–June) of monthly La Quiaca minimum temperatures with sea surface temperatures^33^. The spine δ^18^O shares spatial correlation features with both July–June and January–December spatial field correlations because of its less than annual age-modelling resolution, which may put δ^18^O minimums in the year before or ahead of where they belong, just as a January–December annualization of minimum temperatures from La Quiaca will yield different results in this respect. Bottom panels (D, E, F) are the same but with both isotope and temperature time-series linearly detrended.

The minimum annual values of the dated δ^18^O spine time-series have a significant positive relationship to La Quiaca mean annual temperatures (Fig. 5a; *r* = 0.58; *P* < 0.0001) 330 km to the southeast and a significant negative relationship (Fig. 5b; *r* = −0.41, *P* < 0.002) to the annual mean of breakpoint adjusted (July–June) minimum temperatures from Oruro 250 km to the northeast (Berkeley ID #152744). This makes sense, as the Oruro and La Quiaca minimum temperature records are also negatively correlated (Fig. 5c; *r* = −0.37, *P* < 0.005). Similar to tree-ring proxies of precipitation on the Altiplano (Morales *et al.*, 2012), the dated δ^18^O spine time-series show no significant correlation with annual (July–June) regridded minimum temperature in the same year, but strong and significant negative correlations with that in previous years (Supplemental Data Fig. 3e and 3h; Lag +1, *r* < −0.5 and *P* < 0.001; Lag +2, *r* < −0.4 and *P* < 0.001). This pattern is identical to the relationship of instrumental temperature records at La Quiaca to the same annual (July–June) regridded minimum temperature records (Fig. 6d and 6g). Regardless of how isotope data are treated (i.e. linearly detrended), minimum annual δ^18^O in spines shows a strong positive relationship to the annual mean of minimum monthly temperatures at La Quiaca (i.e. lower annual mean minimum temperatures lead to lower VPD, lower evaporation, and lower δ^18^O values in spines). The temporal and geographic offset in the spatial correlation fields of spine δ^18^O and La Quiaca temperature with the regridded temperature data on the Altiplano is most likely attributable to the sparse and intermittent nature of instrumental data (Garreaud, 2000) on the Altiplano; a feature also seen in nearby instrumental and tree-ring reconstructions of precipitation (Morales *et al.*, 2012).

Farther afield, there is a strong and significant positive relationship between annual minimum spine δ^18^O and July–June mean annual Atlantic SSTs (Fig. 6) (Rayner, 2003). There is also a striking similarity between the spatial correlation fields of the La Quiaca temperature data and SST and spatial correlation fields of the annual minimum δ^18^O of spines and SST. Because annual boundaries in the spine δ^18^O time-series are poorly constrained (i.e. near-annual), the spine δ^18^O record shares many similarities with spatial correlations of La Quiaca temperature when it is calculated as a July–June year or a January–December year (Fig. 6a and 6c). Unlike La Quiaca, however, the spine δ^18^O record appears to be more strongly related to North Atlantic SST near Iceland and in the South Atlantic (Fig. 6b and 6e). We suggest this is the result of the interior of the southern Altiplano being more strongly sheltered from Easterly trade winds than La Quiaca which rests at high elevation but on the south-eastern edge of the Altiplano.

Oxygen isotope ratios in pasacana spines are strongly affected by minimum temperatures, in contrast to North American columnar cactus which respond primarily to precipitation (English *et al.*, 2010b). Spine δ^18^O values are controlled by the isotopic composition of water in the plant, and that is always offset to more positive values due to evaporated water retained in the plant stem. In the American southwest rainfall supply and aridity are the dominant factor affecting spine isotope chemistry. For pasacana on the Altiplano, water supply does not vary greatly from year to year; the SASM (South American Summer Monsoon) reliably recharges cactus water to full capacity each year. Because almost all cactus plants use the Crassulacean acid metabolism (CAM) photosynthetic pathway, they preferentially open their stomata at night (Gibson *et al.*, 1986). As a consequence, transpiration primarily occurs at night and transpiration rates are controlled by night-time temperatures (i.e. minimum temperatures) and vapour pressure deficit (VPD). With the cool/cold night temperatures at ~ 4000 masl, small increases in temperature can have an exponential effect on VPD, leading to higher δ^18^O in spines (i.e. more evaporated cactus stem water) in years with higher minimum (night-time) temperatures.

## Discussion

Previous research has yielded considerable evidence that the sequence of spines arranged along the length of cactus stems may serve as robust climate recorders similar to that of growth rings in long-lived woody species (English et al., 2007, 2010a,b; Hultine et al., 2019). Results from this study expand previous research of isotopic signatures in the spines of the iconic giant saguaro to the pasacana cactus, occurring in one of the most remote regions of the Americas. Results from our analysis indicate that similar to many other cacti species, precipitation is important for this species’ recruitment. Results also reveal a relationship between night-time temperatures on the Altiplano and the variation in oxygen isotope values in spines (δ^18^O). The annual δ^18^O minimums from 58 years of in-series spine tissue from pasacana on the Altiplano provides at least decadal proxy-records of temperature (*r* = 0.58; *P* < 0.0001), and evidence suggests that there are longer records connecting modern Altiplano temperatures to sea-surface temperatures (SSTs) in the Atlantic Ocean. We acknowledge that it is not possible to make strong inferences using isotopic evidence from only one spine series collected from a single individual plant. Nevertheless, to our knowledge this is the first evidence other than in giant saguaro that isotope records in cactus spines correlate with climate variability. The use of radiocarbon and acanthochronology on pasacana cactus yields the best quantitative evidence to date that these cacti may live to be over 400 years old. Further studies using a variety of pasacana age classes would be useful in confirming and refining the age model presented here.

Future scenarios of climate change suggest increasing temperatures on the Altiplano between 0.2° and 0.6°C per decade (Magrin *et al.*, 2014) and we would anticipate consequent impacts on cactus growth, mortality, fitness and recruitment that will be reflected in the isotope signals captured in spine tissues. There may be a threshold where increasing temperatures and consequent increases in plant transpiration lead to an increasing reliance on the SASM and a switch in the isotopic response of cactus spines from temperature sensitive to precipitation sensitive, although at what temperature threshold this occurs is unknown. Plant stress resulting from temperature increases may be exacerbated by predicted decreases in precipitation (Magrin *et al.*, 2014). For the 462 pasacana cactus measured in this study, there was a steep decline in mortality after a pasacana reaches 154 cm (Fig. 3), roughly 50 to 60 years in age (Methods). For the whole population sampled, Silverman tests suggest there are at least 5 transformed modes (i.e. peaks, Supplemental Data Fig. 4a) or 7 non-transformed modes (not shown) in the survivorship curve, although we conservatively estimate 5 peaks. These peaks in recruitment occur during or just after (within 10 years) inferred wet periods present in the tree-ring reconstruction of precipitation developed by Morales *et al.* (2012) (Supplemental Data Fig. 5) for a nearby site. These data do not show whether peaks and troughs in the survivorship curve are related to increased recruitment or increased mortality, however, we hypothesize that extended periods of increased precipitation/decreased temperatures or years without severe drought result in recruitment events that are reflected in peaks in survivorship curves. Demographically, it appears that pasacana: 1) reach maturity (i.e. the addition of arms) at around 100 to 150 years old (Fig. 2b); 2) attain their full stature later than their North American counterparts; and 3) are very long-lived (~400 years old). These estimated ages are the oldest quantitative estimates for a cactus anywhere in the world. Radiocarbon dating of taller and shorter cactus will help to refine the pasacana growth model and allow better age-modelling of isotope time-series derived from pasacana.

A strong link between North Atlantic SST variability and Altiplano climate is implied by palaeoclimate records from the region. Modern climate data across the Altiplano suggests that modern climate variability across the Altiplano is spatially heterogeneous; the data presented here suggest a strong but previously unreported link between Altiplano temperatures and Atlantic SST. For the cactus data, this link is pronounced across the North Atlantic, a situation that cannot be attributed to links between Pacific and tropical Atlantic SSTs (Enfield *et al.*, 1997). Taken together this suggests that temperatures on the Altiplano are not only sensitive to distant global anomalies, but that the dominant mode of variability (ENSO vs. North Atlantic) is today very spatially heterogeneous. Better understanding of this complicated spatial pattern of interannual variability is essential to forecasting future climate across the region.

The δ^18^O minimum variations in pasacana spines have a strong positive relationship to both the minimum annual temperature recorded at a nearby instrumental station and to mean annual Tropical and North Atlantic sea surface temperatures. Today, during the Austral summer, moisture from the Amazon and Chaco lowlands is transported onto the Altiplano when the ITCZ is displaced southward, and a broad band of heavy precipitation extends from the southern half of the Amazon Basin to northern Argentina (Garreaud *et al.*, 2009). At the same time, a deep continental low forms over the Gran Chaco region of Argentina (~25°S), forcing the easterly winds that flow over the Amazon to turn southward, channeling moisture along the eastern slope of the Andes in a low-level jet (Seluchi *et al.*, 2003; Garreaud *et al.*, 2009) and feeding intense convective storms as far south as 35°S (Garreaud *et al.*, 2009). These storms lead to the formation of an upper-level high pressure cell (the Bolivian High) that in turn increases the transport of moisture onto the Altiplano (Lenters *et al.*, 1997). This strong seasonal cycle has been described as the South American Summer Monsoon (SASM) (Zhou *et al.*, 1998; Vera *et al.*, 2006). In general, ENSO exerts a modest influence on internal moisture transport onto the Altiplano, with La Niña years tending to correlate with stronger easterly (trade) winds and more rainfall (Aceituno, 1988; Vuille *et al.*, 1998; Vuille, 1999; Garreaud *et al.*, 2001; Vuille *et al.*, 2004). However, the spatial variability of climate anomalies across the Altiplano is pronounced, with a more significant ENSO influence on the north of the Salar de Uyuni and Coipasa (Vuille *et al.*, 2004) (Fig. 1a, dashed line). No clear link has been observed between modern Atlantic SSTs and central Andean precipitation (Vuille, 2003; Vuille *et al.*, 2003), but the first-order control on Quaternary lake expansion on the Altiplano is North Atlantic cold events (Placzek *et al.*, 2009). Diurnal temperature fluctuations on the Altiplano (~3800 m) are extreme (>12°C). These diurnal changes exceed annual mean monthly temperature variability of ~ 6°C. Moreover, much of the seasonal variability in temperature is driven by variability in minimum (night-time) monthly temperatures. North of the Salars de Uyuni and Coipasa, ENSO exerts a modest influence on temperature, with La Niña years (also generally wet) tending to correlate with lower temperatures. In the southern Altiplano, temperature records are dominated by a broad warming since ~ 1970.

The relationship between spine δ^18^O, Altiplano temperatures (and that temperature’s impact on evaporation) and north Atlantic SST bolsters previous work that finds strong relationships between southern Altiplano lake cycles, driven by the balance of precipitation and evaporation, and north Atlantic SST in the late Pleistocene(Placzek *et al.*, 2013). There also appears to be a sharp divide between the southern and northern Altiplano with respect to the influence of north Atlantic SST, a divide that straddles large columnar cactus populations (e.g. to the north of Salar de Uyuni *Echinopsis tarijensis* (Vaupel) Friedrich & G.D. Rowley is common) and raises the question, ‘Can the movement of this divide be mapped through time using cactus spines or demography?’ In addition, statistical analyses of minimum spine δ^18^O in packrat middens, often filled with cactus spines (Supplemental Data Fig. 6), may also be used to determine temperature relationships to north Atlantic SST in South America beyond the lifetime of a cactus.

Results from this study extend the growing body of evidence that isotopic signals in the spines of giant cacti reflect their sensitivity to climate conditions as a function of stem water balance and photosynthetic processes (English *et al*., 2007; Williams *et al*., 2014; Hultine *et al*., 2019). Although most previous research on the relationship between spine isotopic signals and climate has been conducted on the iconic saguaro cactus (English *et al*., 2007, 2010a,b; Hultine *et al*., 2018), we now have strengthening evidence that these isotopic approaches can be universally applied to study giant cacti sensitivity to climate variation and extremes. Importantly, cacti are among the most threatened taxonomic groups on the planet with over 30% of all species of cacti considered threatened or endangered (Goettsch *et al*., 2015), including the pasacana cactus studied here. Therefore, the methods of acanthochronology presented here are not just applicable to the Altiplano but can be utilized across the Americas to better understand the sensitivity of cacti species to their environment and to better ensure their survival while also informing current models of climate variability in regions with few instrumental or paleoclimate proxies.

## Funding

This research was funded by the National Geographic Society Committee for Research and Exploration (NGS#8792–10), the Australian Institute of Nuclear Science and Engineering (ALNGRA#12135), and the National Science Foundation (Grant #IOS 0717395 and #IOS 0717403).

## Author contributions

N.B.E., D.G.W., M.M. collected samples in the field. N.B.E., D.M. performed the stable isotope sample preparation and carbon analyses and also the climate correlation analyses. D.L.D. performed the oxygen stable isotope analyses. Q.H. performed the radiocarbon analysis and OxCal ^14^C age models. N.B.E. wrote the paper with comments and input provided by all other authors with K.R.H providing significant contributions and editing.

## Supplementary Material

Supplemental_Data_(v26)_coaa123
