# Supplementary material for: Age-growth relationships, temperature sensitivity and palaeoclimate-archive potential of the threatened Altiplano cactus Echinopsis atacamensis
Source: Conserv Physiol. 2021 Jan 27;9(1):coaa123. doi: 10.1093/conphys/coaa123 (PMC7805519; doi:10.1093/conphys/coaa123)
Supplement: Supplemental_Data_(v26)_coaa123 [file supplemental_data_(v26)_coaa123.pdf]

Supplemental Data for "Age-growth relationships, temperature sensitivity and palaeoclimate-archive potential of the threatened Altiplano cactus *Echinopsis atacamensis*"

English, NB[1*], DL Dettman[2,3], Q Hua[4], JM Mendoza[5], D Michelle[6], KR Hultine[7], DG Williams[8]

[1] School of Health, Medical and Applied Science, Central Queensland University, 538 Flinders St West, Townsville, QLD 4810, Australia

[2] Department of Geosciences, University of Arizona, Tucson, AZ 85721, USA

[3] Estuary Research Center, Shimane University, Matsue, 690-8504, Japan

[4] Australian Nuclear Science and Technology Organisation, Lucas Heights, NSW 2234, Australia

[5] Museo de Historia Natural Noel Kempff Mercado, Casilla 2489, Santa Cruz, Bolivia

[6] Murdoch University, Perth, WA 6009, Australia

[7]Department of Research, Conservation and Collections, Desert Botanical Garden, Phoenix, AZ 85008, USA

[8] Department of Botany, University of Wyoming, Laramie, WY 82071, USA

*Author to whom correspondence should be addressed

n.english@cqu.edu.au

Supplemental Data Table 1. Radiocarbon results of 18 spine samples from 2 cactuses (IP19 and IP20).[1-3]

	Lab ID	Sample ID	Depth (cm)[a]	Height (cm)[b]	$\delta^{13}C$ (‰)	$F^{14}C \pm 1\sigma$		Unmodelled cal. AD 68.2% conf. level		95.4% conf. level		Mean	1σ	Median	Simple Sequence - Modelled cal. AD[c] 68.2% conf. level		95.4% conf. level		Mean	1σ	Median
		Top	0	495				2011	2011	2011	2011	2011	0	2011	2011	2011	2011	2011	2011	0	2011
1	OZP281	IP19-30	30	465	-11.8	1.0826	0.0032	2002	2004	1957	2004	1998	14	2003	2002	2004	2001	2004	2003	1	2003
2	OZP282[d]	IP19-50	50	445	-	-	-														
3	OZP283	IP19-99	99	396	-11.7	1.1768	0.0036	1959	1989	1959	1989	1979	13	1988	1987	1989	1987	1990	1988	1	1988
4	OZP284	IP19-150	150	345	-12.8	1.4182	0.0034	1973	1974	1963	1974	1972	4	1974	1973	1974	1973	1974	1974	1	1974
5	OZP285	IP19-200	200	295	-10.7	1.5842	0.0035	1967	1968	1967	1968	1967	1	1967	1967	1968	1964	1968	1967	1	1967
6	OZP286	IP19-251	251	244	-9.5	1.1890	0.0030	1960	1988	1959	1989	1976	13	1986	1960	1962	1959	1963	1961	1	1961
7	OZP287	IP19-299	299	196	-10.6	0.9723	0.0029	1667	1798	1652	1954	1752	60	1758	1951	1954	1949	1955	1952	2	1952
8	OZP288	IP19-353	353	142	-10.0	0.9748	0.0029	1669	1953	1656	1955	1764	70	1759	1948	1953	1937	1954	1948	6	1950
9	OZP289	IP19-410	410	85	-13.3	0.9790	0.0036	1677	1951	1671	1955	1811	87	1821	1939	1951	1923	1953	1940	11	1945

Indices: A_{model} = 62.9%, $A_{overall}$ = 62.3%

	Lab ID	Sample ID	Depth (cm)[a]	Height (cm)[b]	$\delta^{13}C$ (‰)	$F^{14}C \pm 1\sigma$		Unmodelled cal. AD 68.2% conf. level		95.4% conf. level		Mean	1σ	Median	Simple Sequence - Modelled cal. AD[c] 68.2% conf. level		95.4% conf. level		Mean	1σ	Median
		Top	0	495				2011	2011	2011	2011	2011	0	2011	2011	2011	2011	2011	2011	0	2011
10	OZP290	IP20-25	25	470	-10.6	1.0705	0.0062	2004	2008	1957	2010	2001	14	2005	2004	2007	2003	2010	2006	2	2006
11	OZP291	IP20-60	60	435	-11.5	1.0919	0.0029	2000	2002	1958	2003	1999	9	2001	2000	2002	2000	2003	2001	1	2001
12	OZP292	IP20-99	99	396	-11.2	1.0943	0.0036	2000	2002	1999	2003	1999	8	2000	1999	2001	1999	2002	2000	1	2000
13	OZP293	IP20-150	150	345	-10.9	1.1331	0.0031	1993	1994	1958	1995	1989	11	1993	1993	1994	1992	1995	1993	1	1993
14	OZP294	IP20-200	200	295	-12.1	1.2436	0.0036	1982	1983	1962	1984	1980	6	1982	1982	1983	1982	1984	1982	1	1982
15	OZP295	IP20-250	250	245	-14.0	1.3577	0.0034	1976	1976	1963	1977	1974	4	1976	1976	1976	1975	1977	1976	1	1976
16	OZP296	IP20-300	300	195	-10.2	1.4727	0.0035	1972	1972	1963	1973	1971	2	1972	1972	1972	1971	1973	1972	1	1972
17	OZP297	IP20-350	350	145	-9.0	1.5729	0.0038	1967	1968	1964	1968	1967	2	1967	1967	1968	1964	1968	1967	2	1968
18	OZP298[e]	IP20-430	430	65	-10.0	1.1200	0.0033	1994	1996	1958	1997	1990	12	1995	1958	1959	1958	1959	1959	1	1959
					-10.1	1.1262	0.0030														
						1.1234	***0.0031***														

Indices: A_{model} = 70.3%, $A_{overall}$ = 70.2%

Note :

[a] - Distance from cactus apex

[b] - Approximate distance from cactus base

[c] - Calendar ages of spines from each cactus were modeled using the OxCal simple sequence model (Bronk Ramsey, 2008) with the date of sampling (August, 2011) as the top boundary, and the Southern Hemisphere Zone 1-2 bomb data (Hua et al., 2013) extended back in time by the SHCal13 calibration curve (Hogg et al., 2013)

[d] - Sample was accidentally lost during sample preparation

[e] - Two fractions of sample OZP298 were analysed. The 1st fraction was "spike" and the other one was "fibre". The $F^{14}C$ value in bold-italic was the weighted mean of the two analyses.

Supplemental Data Table 2. Polynomials for the saguaro and pasacana growth models.[4]

Polynomial	Saguaro SNPE	Pasacana (IP19)	Pasacana (IP20)
a	-0.222287	-0.30	-0.30
b	1.0	0.23	0.32
c	0.606605	0.90	0.90
d	-0.0000353372	-0.00013	-0.00013

Supplemental Data Figure 1: ^{14}C and height data from spines on two pasacana cactus. The line is the best fit model using the Drezner growth model for saguaro. The misfit can be clearly seen, even when an appropriate adjustment factor is used to adjust the saguaro model to fit the pasacana data.

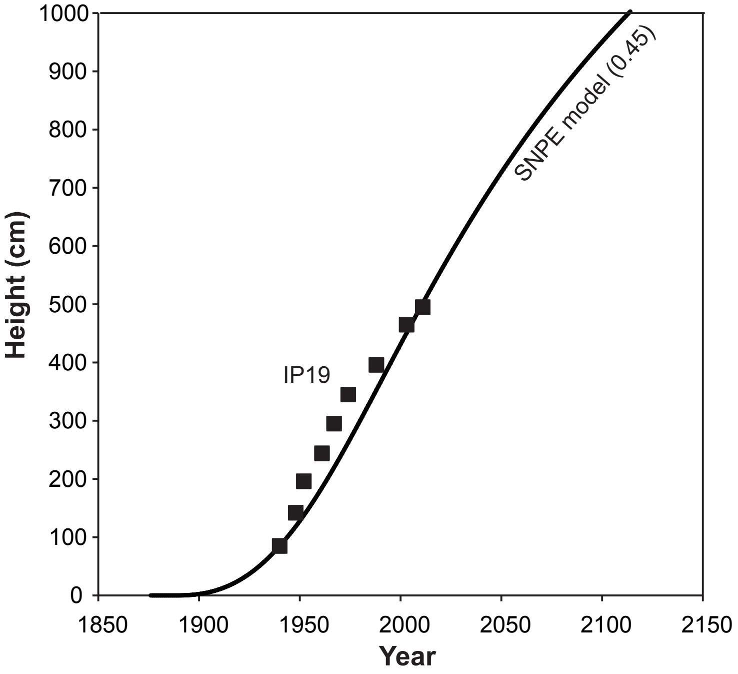

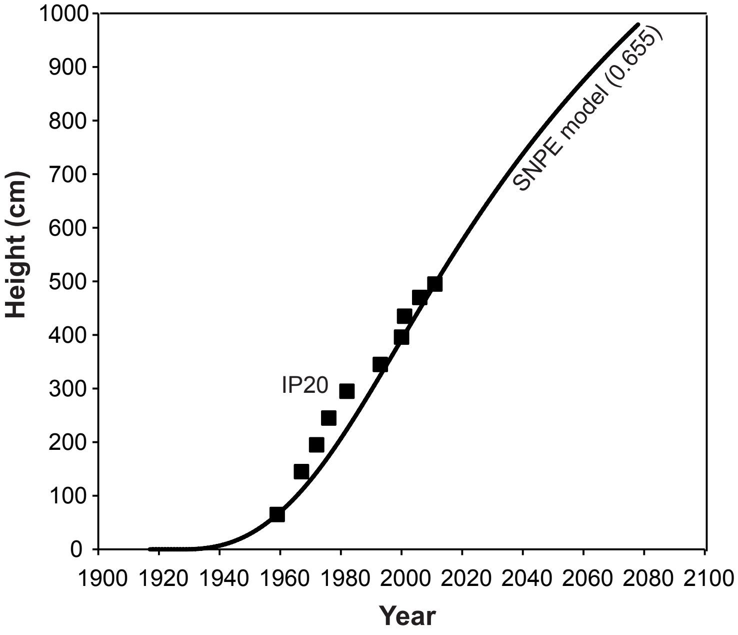

Supplemental Data Figure 2:
Comparison of mean pasacana (black) and saguaro (gray) growth curves. For saguaro, *b* = 0.55, or 55% of the growth rate of the Saguaro National Park East saguaro population.[4]

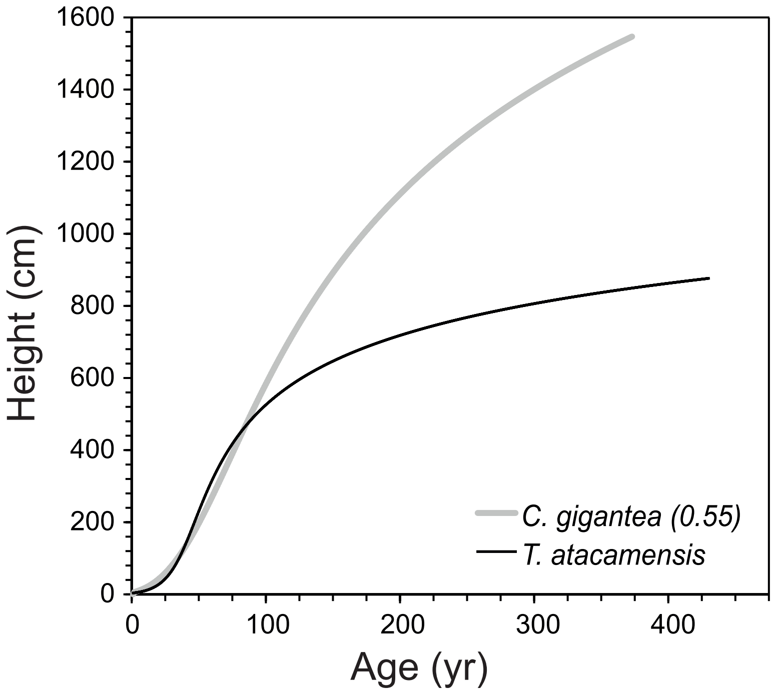

Supplemental Data Figure 3. Spatial correlation fields of annual means of monthly regridded temperature minimums (T_{min} CRU 3.21) and annual means of monthly La Quiaca minimum temperatures (A, D, G) and annual minimum $\delta^{18}O$ of spine tissue (B, E, H) between 1956 and 2010. The spatial correlation fields of linearly detrended annual means of CRU and spine $\delta^{18}O$ are also shown (C, F, I). Middle and bottom panels show the spatial field correlations of a +1 and +2 year lag, respectively. Only significant correlations are shown (α = 0.05).

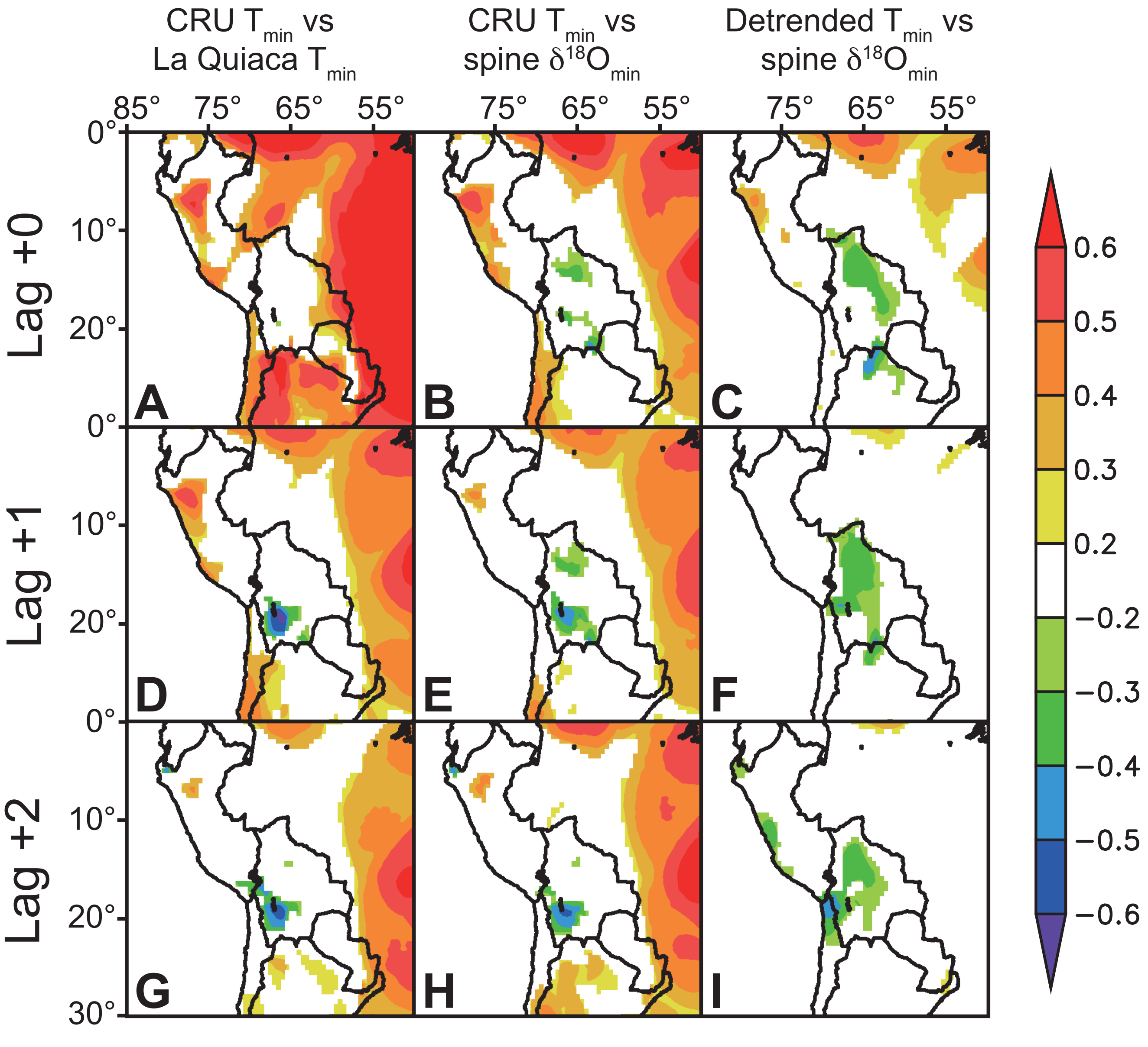

Supplemental Data Figure 4: Kernel density plots of multimodal analysis using Silverman's test (a = 0.05) for height data (A) and natural-log transformed height data (B). Bottom middle panel in (A) is shown in Figure 3 of the text.

a

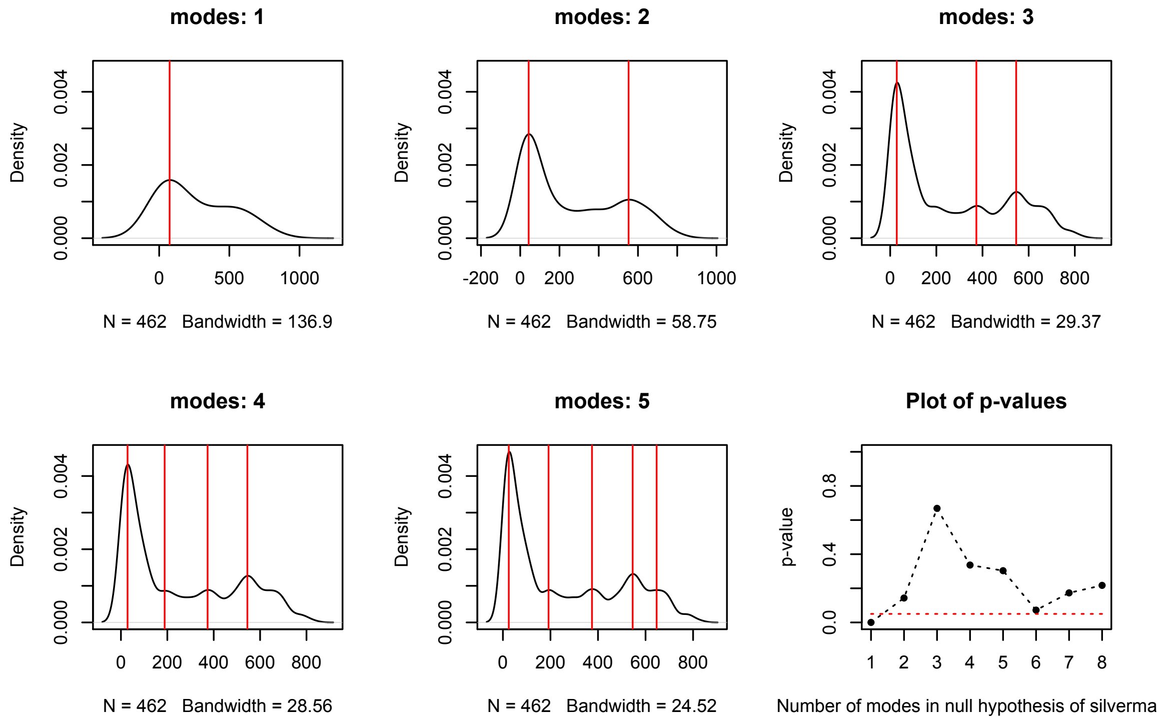

b

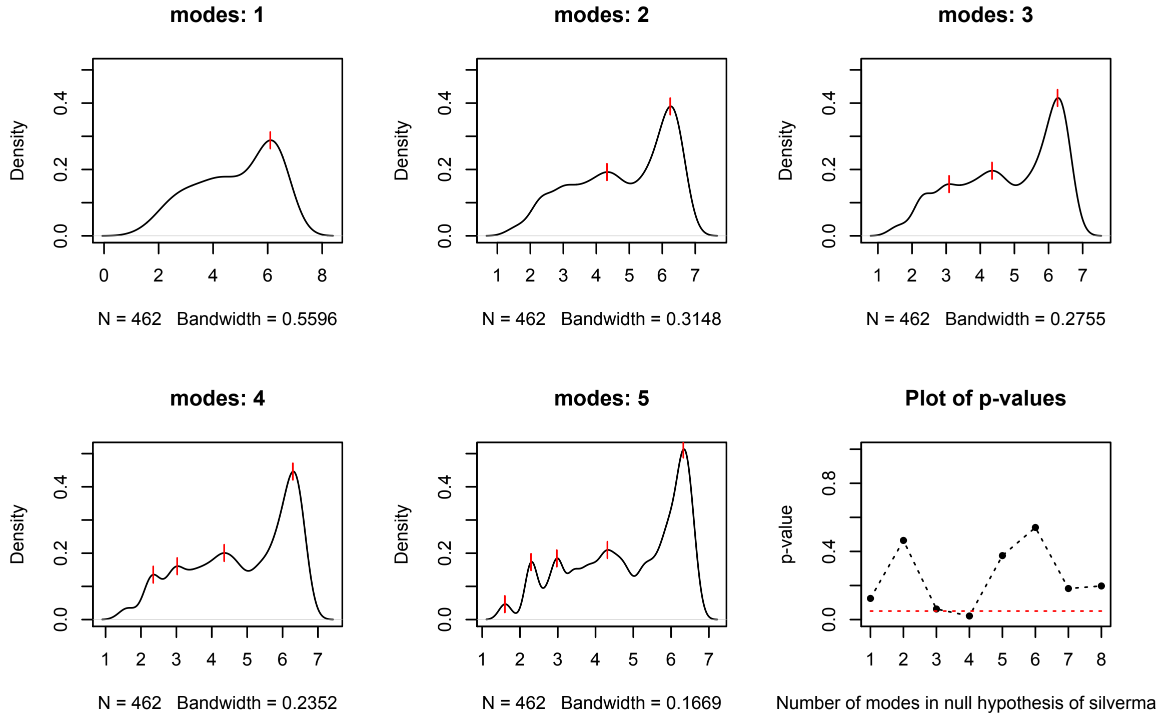

Supplemental Data Figure 5: Modal peaks from survivorship curve overlain on Figure 4 from Morales et al.[5]. The Morales et al. figure shows November-October precipitation reconstruction (black) from *Polylepis tarapacana* on the Altiplano (thick line is 35 yr cubic smoothing spline set at 50% of variance with red and blue indicating above and below the mean, respectively). The light blue dashed line represents an insignificant mode at roughly 792 cm in Figure 3 of the text.

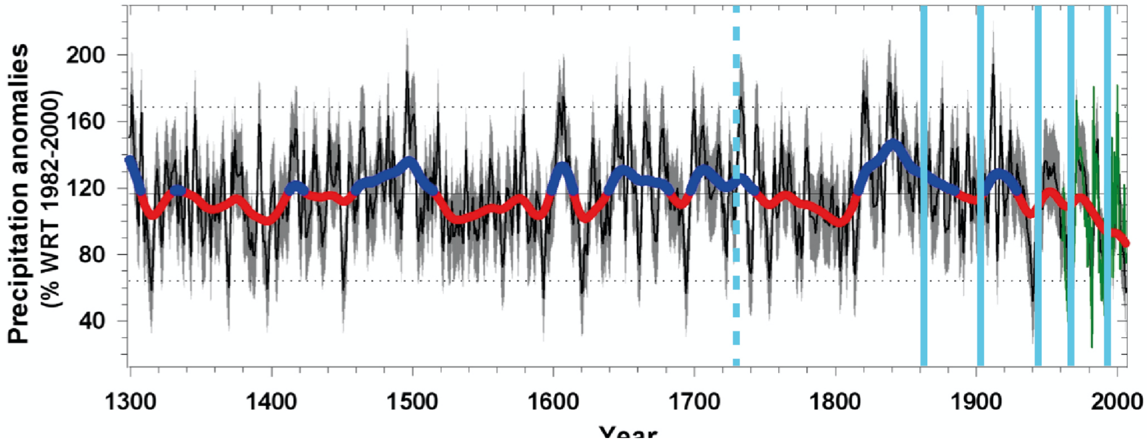

Supplemental Data Figure 6. Oreocereus spines (identified only to genus) in a ~600 year-old packrat midden from Quebrada La Higuera (near Arica, Chile). Photo courtesy of Dr. Claudio Latorre.

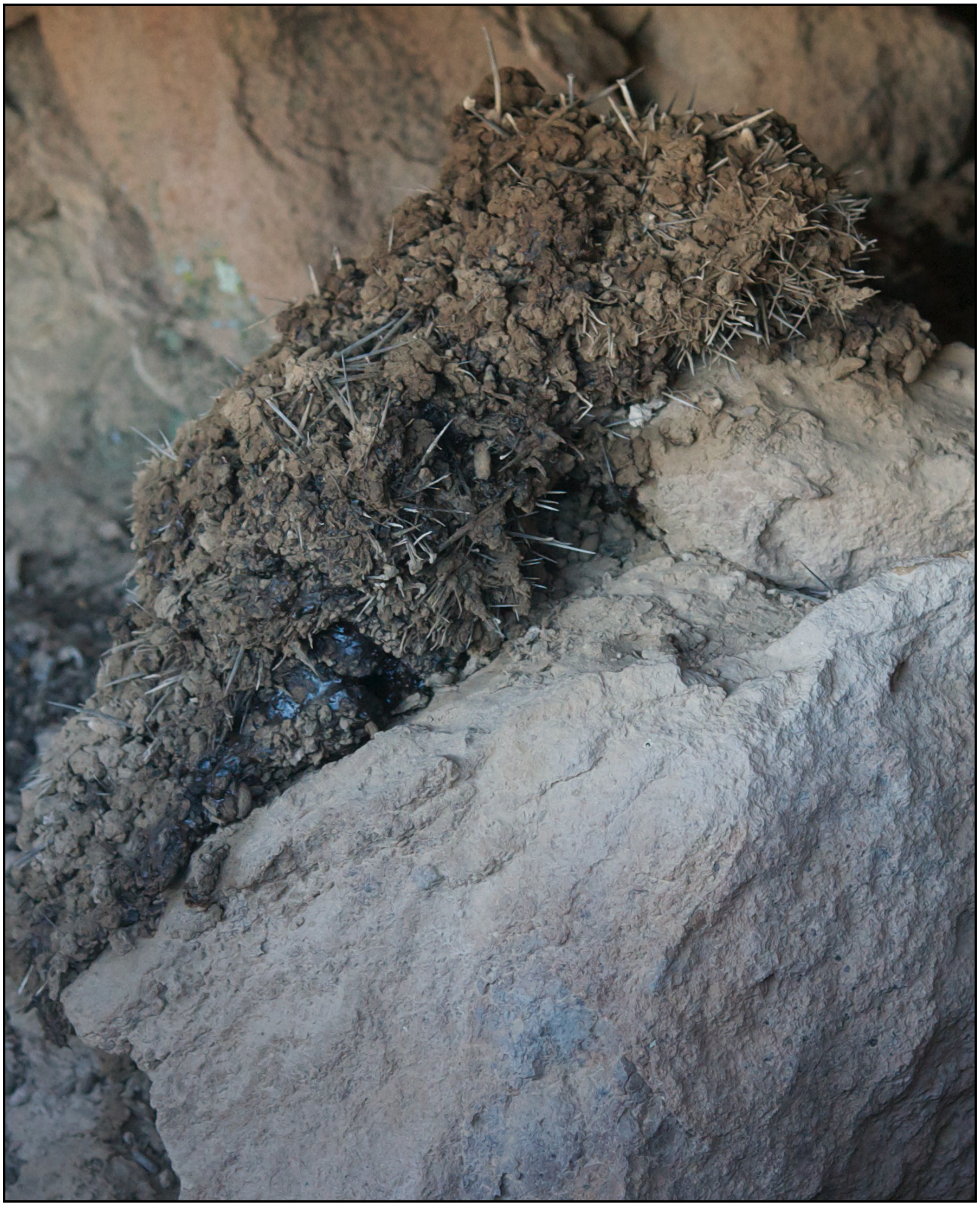

Supplemental Data: Stable isotope data
All measurements are from the apex of the cactus (i.e. 0 cm = cactus apex = spine depth)

IP 19 raw d13C data (Max C = -8.13‰, Min C = -13.04‰, Mean C = -10.37‰, n = 224)

[^{14}C spine depth (cm), ^{14}C age (year AD), $\delta^{13}C$ spine depth (cm), $\delta^{13}C$]

```
0,2011,0,-10.75
30,2003,3,-12.28
99,1988,4,-11.56
150,1974,6,-11.03
200,1967,8,-10.35
251,1961,9,-10.8
299,1952,11,-10.41
353,1948,13,-10.5
410,1940,14,-11.66
,,16,-11.65
,,18,-11.16
,,19,-10.95
,,20,-10.73
,,21,-11.38
,,22,-12.03
,,24,-11.41
,,25,-10.73
,,27,-12.26
,,28,-11.65
,,30,-11.43
,,31,-11.61
,,33,-10.99
,,35,-11.16
,,37,-10.72
,,39,-11.63
,,40,-12.03
,,42,-11.2
,,43,-12.88
,,45,-11.69
,,47,-13.04
,,49,-10.81
,,50,-9.99
,,52,-11.36
,,54,-11.45
,,55,-11.83
,,57,-10.64
,,58,-11.07
,,60,-12.01
,,61,-11.11
,,62,-12.73
,,63,-11.52
,,65,-11.79
,,67,-11.49
,,69,-10.84
,,70,-11.39
,,73,-11.36
,,74,-11.09
```

```
,,76,-10.97
,,78,-12.08
,,80,-11.26
,,82,-10.52
,,83,-11.81
,,85,-10.48
,,87,-10.92
,,89,-10.88
,,90,-11.06
,,92,-12.3
,,93,-12.75
,,95,-11.62
,,96,-10.97
,,98,-11.01
,,99,-11.06
,,101,-11.5
,,102,-10.38
,,103,-10.37
,,105,-10.36
,,107,-11.99
,,109,-11.9
,,110,-11.4
,,112,-11.29
,,113,-10.64
,,114,-11.01
,,115,-10.31
,,117,-10.25
,,119,-10.11
,,121,-10.91
,,122,-10.73
,,124,-10.33
,,125,-10.96
,,126,-9.66
,,128,-9.99
,,130,-11.34
,,132,-11.24
,,134,-9.81
,,135,-9.81
,,137,-10.21
,,138,-9.36
,,140,-9.08
,,143,-10.79
,,144,-10.08
,,146,-11.06
,,148,-10.32
,,150,-10.53
,,152,-10.45
,,154,-11.04
,,155,-10.71
,,157,-10.47
,,159,-10.03
,,161,-10.42
,,162,-10.26
,,163,-9.26
,,165,-9.85
```

```
,,166,-9.72
,,168,-9.46
,,169,-10.36
,,170,-11.52
,,172,-10.72
,,173,-10.53
,,174,-11.13
,,176,-10.39
,,177,-10.87
,,178,-10.29
,,181,-10.8
,,183,-10.84
,,184,-9.33
,,186,-9.74
,,188,-9.9
,,190,-10.54
,,192,-9.73
,,194,-10.23
,,196,-10.23
,,198,-9.28
,,200,-10.74
,,202,-9.85
,,204,-10.07
,,206,-9.3
,,208,-10.48
,,210,-9.98
,,211,-10.08
,,213,-11.19
,,214,-8.75
,,216,-10.44
,,219,-10.57
,,222,-9.86
,,224,-8.56
,,225,-9.66
,,227,-9.55
,,229,-10.12
,,230,-10.48
,,232,-9.88
,,234,-9.73
,,236,-10.23
,,238,-9.74
,,239,-9.8
,,241,-10.11
,,243,-8.22
,,245,-8.44
,,247,-8.68
,,249,-10.13
,,251,-10.14
,,253,-9.2
,,255,-10.52
,,257,-9.39
,,259,-8.69
,,262,-10.04
,,264,-10.19
,,266,-11.01
```

```
,,269,-9.3
,,271,-8.13
,,273,-8.62
,,275,-8.88
,,278,-9.09
,,279,-9.86
,,282,-10.51
,,284,-9.4
,,287,-8.98
,,289,-10.37
,,291,-10.14
,,293,-10.11
,,296,-8.96
,,299,-10.11
,,301,-9.72
,,304,-9.96
,,306,-9.88
,,308,-9.34
,,310,-9.46
,,313,-9.16
,,316,-8.89
,,318,-10.2
,,321,-8.98
,,324,-9.94
,,326,-10.16
,,329,-8.92
,,331,-9.81
,,334,-9.72
,,337,-9.52
,,339,-9.77
,,342,-9.49
,,345,-9.62
,,348,-8.71
,,351,-10.74
,,353,-9.62
,,356,-9.23
,,359,-10.14
,,364,-9.68
,,367,-10.28
,,368,-9.81
,,371,-8.81
,,374,-9.55
,,377,-10.12
,,380,-9.36
,,382,-10.26
,,384,-10.82
,,386,-10.32
,,388,-10.49
,,391,-10.12
,,393,-10.48
,,395,-9.7
,,397,-9.63
,,400,-10.19
,,402,-10.37
,,405,-9.89
```

```
,,407,-10.01
,,410,-9.24
,,412,-9.42
,,414,-9.87
,,416,-9.72
,,418,-9.94
,,421,-9.7
,,424,-9.8
,,426,-9.43
,,429,-10.01
,,431,-9.04
,,435,-9.89
```

IP 19 raw $\delta^{18}O$ data (Max O = 62.47‰, Min O = 41.63‰, Mean O = 52.03‰,)

[^{14}C spine depth (cm), ^{14}C age (year AD), $\delta^{18}O$ spine depth (cm), $\delta^{18}O$]

```
0,2011,0,49.45
30,2003,3,46.58
99,1988,4,54.54
150,1974,6,57.31
200,1967,8,49.2
251,1961,9,58.73
299,1952,11,52.35
353,1948,13,55.44
410,1940,14,54.54
,,16,55.55
,,18,54.29
,,20,58.89
,,21,59.15
,,22,59.48
,,24,56.66
,,25,62.47
,,27,58.48
,,28,56.55
,,30,58.87
,,31,60.07
,,33,58.33
,,35,57.12
,,37,60.77
,,39,54.4
,,40,58.98
,,42,61.71
,,43,55.57
,,45,59.24
,,47,55.5
,,49,47.58
,,50,50.29
,,52,59.02
,,54,53.75
,,55,60.03
,,57,61.25
,,58,51.65
,,60,56.48
```

,,61,57.3
,,62,58.94
,,63,49.75
,,65,50.48
,,67,51.2
,,69,51.35
,,70,56.32
,,73,48.13
,,74,48.8
,,76,49.22
,,78,54.89
,,80,59.56
,,82,53.56
,,83,51.13
,,85,56.8
,,87,52.83
,,89,49.45
,,90,49.38
,,92,54.81
,,93,53.13
,,95,54.65
,,96,49.74
,,98,54.19
,,99,55.15
,,101,50.54
,,102,57.53
,,103,52.58
,,105,52.48
,,107,42.13
,,109,44.97
,,110,54.1
,,112,41.63
,,113,46.68
,,114,48.73
,,115,47.91
,,117,53.5
,,119,53.93
,,121,51.19
,,122,53.62
,,124,51.36
,,125,49.3
,,126,53.68
,,128,49.82
,,130,47.75
,,132,49.77
,,134,48.66
,,135,49.88
,,137,47.71
,,138,51.64
,,140,55.35
,,143,46.55
,,144,46.18
,,146,42.37
,,148,50.92
,,150,46.2

,,152,46.27
,,154,43.87
,,155,47.56
,,157,44.7
,,159,46.77
,,161,46.96
,,162,49.62
,,163,51.15
,,165,48.7
,,166,54.04
,,168,52.49
,,169,54.43
,,170,51.93
,,172,49.51
,,173,48.34
,,174,48.55
,,176,49.2
,,177,49.64
,,178,45.65
,,181,46.84
,,183,47.69
,,184,57.34
,,186,58.32
,,188,56.34
,,190,49.84
,,192,52.73
,,194,48.64
,,196,46.87
,,198,50.06
,,200,44.15
,,202,43.59
,,204,44.05
,,206,53.71
,,208,46.87
,,210,49.57
,,211,51.12
,,213,47.71
,,214,55.69
,,216,46.93
,,219,49.57
,,222,53.06
,,224,57.3
,,225,50.04
,,227,55.8
,,229,52.88
,,230,46.24
,,232,45.04
,,234,54.58
,,236,47.74
,,238,48.54
,,239,47.39
,,241,45.56
,,243,53.95
,,245,54.85
,,247,57.4

```
,,249,45.64
,,251,46.14
,,253,57.52
,,255,43.49
,,257,56.33
,,259,60.83
,,262,48.7
,,264,47.14
,,266,48.81
,,269,56.64
,,271,50.84
,,273,59.27
,,275,60.37
,,278,55.29
,,279,54.57
,,282,54.67
,,284,52.64
,,287,53.62
,,289,52.75
,,291,46.19
,,293,49.89
,,296,55.54
```

Supplementary Information: : MATLAB code for transforming cactus data to "annual" time series.

______________.m file__________

```
%Cactus Age Model
% This function use a combination of radiocarbon dates and subjective
% pinned years to convert a cactus isotope record from height to age.
% itime  = input time markers (i.e. tie points) (1 x p)
% idepth = corresponding input depth marks for those tie points (1 x p)
% depth  = the depth that every measurement in 'data' was made (1 x m)
% data   = the data we are age modeling (1 x m)
% step   = the constant time steps between each otime.  (i.e. 1/12) (1 x 1)

close
clear

%%%%%%%%%%%%%%%%%%%%%%%%%%%%%%%%%%%%%%%%%%%%%%%%%%%%%%%%%%%%%%%%%%%%%%%%%%%%
%Apply age model for carbon to d13C data
fls = dir('*pinned_d13c.csv'); %create a structure of all the sites using all file names ending in "pinned"

for f = 1:size(fls,1); %Calculate different variables for each site, one site at a time per loop

%Import data from site specific data files
all = importdata(fls(f).name);
idepth = all(isfinite(all(1:end,1)),1); %height from apex of cactus for itime data
itime = all(isfinite(all(1:end,2)),2); %input time markers (i.e. tie points) (1 x p), for the first pass, this will be 14c only
```

```
depth = all(isfinite(all(1:end,3)),3); %distance from apex of cactus of geochemical data in data
data = all(isfinite(all(1:end,4)),4); %geochemical data of the spine
step = (1/4);       % Divides years into time steps equal to the denominator, this can be changed
                    % to provide higher or lower resolution time-series depending on the growth
                    % rate of the cactus. Faster growing plants (i.e. more spines per year) can use
                    % a higher step number (e.g. 8) as this will not overrepresent the the actual
                    % resolution of a cactus growing 10 spines per year. Also, it should be
                    % considered that many cactus do not grow year round, and this program
                    % represents it as such. Caveat emptor...

[otime,odata,otrough] = agemodel(itime, idepth, depth, data, step); %use the function agemodel
                                                                     %to produce an agemodeled
geochemical data series
end

max = max(otime);
min = min(otime);
annualrange = ((max-min)+1);
size1 = size(otime,1);

agemodelleddata = zeros(size(odata),5);
agemodelleddata(:,1) = otime;
agemodelleddata(:,2) = odata;

% Account for Suess effect (from McCarroll and Loader)
% Francey et al. (1999) have compiled a high precision record of
% atmospheric d13C based on Antarctic ice cores which, for the purposes of
% correcting tree-ring data, can be summarised by two straight segments
% between 1850 and 1961, with an annual decline of 0.0044% and between 1962
% and 1980 with a steeper annual decline of 0.0281%.

c=0;  %Count how many time-steps are post 1962
for f=1:(size(otime));
   if otime(f,1)>1961.9999;
      c=c+1;
   end
end

% Subtract Suess effect from data depending on whether the data is post or
% pre-1962.
for f=1:(size(otime));
   if otime(f,1) > 1962;
      agemodelleddata(f,3) = odata(f,1)-((0.0281*step)*(f-1));
   else
      agemodelleddata(f,3) = odata(f,1)-(((0.0281*step)*c)+(0.0044/12)*((f-c)-1));
   end
end


rawdata = nan(size(depth,1),3);
rawdata(:,1) = depth;
rawdata(:,2) = data;
```

```
%%%%%%%%%%%%%%%%%%%%%%%%%%%%%%%%%%%%%%%%%%%%%%%%%%%%%%%%%%%%%%%%%%%%%%%%%%%%%%%%%%%%%%%%%%%%%%%%%%%%%%%%%%%%%%%%%%%%%%%%%%%%
%%%%%%%%%%%%%%%%%%%%
%Apply age model for carbon to d18O data
fls = dir('*pinned_d18o.csv'); %create a structure of all the sites using all file names ending in
"pinned"


for f = 1:size(fls,1); %Calculate different variables for each site, one site at a time per loop
%Import data from site specific data files
all = importdata(fls(f).name);
idepth = all(isfinite(all(1:end,1)),1); %height from apex of cactus for itime data
itime = all(isfinite(all(1:end,2)),2); %input time markers (i.e. tie points) (1 x p), for the first pass,
this will be 14c only
depth = all(isfinite(all(1:end,3)),3); %distance from apex of cactus of geochemical data in data
data = all(isfinite(all(1:end,4)),4); %geochemical data of the spine
%Note that "step" is defined above in the d13c section
[otime,odata,otrough] = agemodel(itime, idepth, depth, data, step); %use the function agemode
                                              %to produce an agemodeled geochemical data series
end

size2 = size(otime,1);

agemodelleddata(:,4) = otime;
agemodelleddata(:,5) = odata;

rawdata(1:(size(data,1)),3) = data;

dlmwrite('IP19_agemodelled_data_d13C_d18O.csv',agemodelleddata,'precision', 6);

%%%%%%%%%%%%%%%%%%%%%%%%%%%%%%%%%%%%%%%%%%%%%%%%%%%%%%%%%%%%%%%%%%%%%%%%%%%%%%%%%%%%%%%%%%%%%%%%%%%%%%%%%%%%%%%%%%%%%%%%%%%%
%%%%%%%%%%%%%%%%%%%%

title = {'Raw IP 19 d13C and d18O'};

minc = floor(nanmin(rawdata(:,2)));
maxc = ceil(nanmax(rawdata(:,2)));
mino = floor(nanmin(rawdata(:,3)));
maxo = ceil(nanmax(rawdata(:,3)));
minh = 0;
maxh = nanmax(rawdata(:,1));


figure(1)
set(gcf,'units','centimeters','position',[1 1 29 5],'paperunits','centimeters',...
    'papersize',[31 11],'paperposition',[0.63 0.63 29 10])

[AX,H1,H2] = plotyy(rawdata(:,1),rawdata(:,2),rawdata(:,1),rawdata(:,3));

%     set(get(AX(1),'Ylabel'),'String','\delta^1^3C');
%     set(AX(1),'Ycolor',[0 0 0]);
```

```
%     set(H1,'LineWidth',1,'color',[0 0 0]);
%     set(get(AX(2),'Ylabel'),'String','\delta^1^8O');
%     set(AX(2),'Ycolor',[0.5 0.5 0.5]);
%     set(H2,'LineWidth',1,'color',[0.5 0.5 0.5]);
    set(get(AX(1),'Ylabel'),'String','\delta^1^3C','fontsize',18);
    set(AX(1),'Ycolor',[0 0 0],'fontsize',12);
    set(H1,'LineWidth',1,'color',[0 0 0]);

    set(get(AX(2),'Ylabel'),'String','\delta^1^8O','fontsize',18);
    set(AX(2),'Ycolor',[0.5 0.5 0.5],'fontsize',12);
    set(H2,'LineWidth',1,'color',[0.5 0.5 0.5]);

    set(AX(1),'YLim',[minc maxc]);
    set(AX(1),'YTick',[minc:2:maxc]);

    set(AX(2), 'xdir','reverse')
    set(AX(2),'XLim',[minh maxh]);
    set(AX(2),'XTick',[minh:40:maxh]);

    set(AX(2),'YLim',[mino maxo]);
    set(AX(2),'YTick',[mino:4:maxo]);

    set(AX(1), 'xdir','reverse')
    set(AX(1),'box','on')
    xlabel('Height (cm)');
    xlabh = get(gca,'XLabel');
    set(xlabh,'Position',get(xlabh,'Position') - [0 .01 0]);
    set(gca,'XLim',[minh maxh]);
    set(gca,'XTick',[minh:20:maxh]);
%     hold on
    set(gca,'XTick',[min:1:max]);
    set(gca,'XTickLabel',' ');

print('-dpdf',[title{1}]);


%%%%%%%%%%%%%%%%%%%%%%%%%%%%%%%%%%%%%%%%%%%%%%%%%%%%%%%%%%%%%%%%%%%%%%%%%%%%%%%%%%%%%%%%%%%%%%%%%%%%%%%%%%%%%%%%%%%%%%%%%%%%%%%%%%%%%%%%%%
%%%%%%%%%%%%%%%%%%%%%%%%%%%%%%%%%%%%%%%%%%%%%%%%%%%%%%%%%%%%%%%%%%%%%%%%%%%%%%%%%%%%%%%%%%%%%%%%

title = {'Age Modelled IP 19 d13C and d18O'};

minc = floor(nanmin(agemodelleddata(:,3)));
maxc = ceil(nanmax(agemodelleddata(:,3)));
mino = floor(nanmin(agemodelleddata(:,5)));
maxo = ceil(nanmax(agemodelleddata(:,5)));

figure(2)
set(gcf,'units','centimeters','position',[1 1 29 5],'paperunits','centimeters',...
    'papersize',[31 11],'paperposition',[0.63 0.63 29 10])

[AX,H1,H2] =
```

```
plotyy(agemodelleddata(:,1),agemodelleddata(:,3),agemodelleddata(:,4),agemodelleddata(:,5));


    set(get(AX(1),'Ylabel'),'String','\delta^1^3C','fontsize',18);
    set(AX(1),'Ycolor',[0 0 0],'fontsize',12);
    set(H1,'LineWidth',1,'color',[0 0 0]);

    set(get(AX(2),'Ylabel'),'String','\delta^1^8O','fontsize',18);
    set(AX(2),'Ycolor',[0.5 0.5 0.5],'fontsize',12);
    set(H2,'LineWidth',1,'color',[0.5 0.5 0.5]);

    set(AX(1),'YLim',[minc maxc]);
    set(AX(1),'YTick',[minc:2:maxc]);

    set(AX(2),'XLim',[min max]);
    set(AX(2),'XTick',[min:10:max]);

    set(AX(2),'YLim',[mino maxo]);
    set(AX(2),'YTick',[mino:4:maxo]);



    set(AX(1),'box','on')
    xlabel('Year');
    set(gca,'XLim',[min max]);
    set(gca,'XTick',[min:10:max]);
    hold on
    set(gca,'XTick',[min:1:max]);
    set(gca,'XTickLabel',' ');

print('-dpdf',[title{1}]);

%%%%%%%%%%%%%%%%%%%%%%%%%%%%%%%%%%%%%%%%%%%%%%%%%%%%%%%%%%%%%%%%%%%%
%%%%%%%%%%%%%%%%%%%%%%
% Determine max, min and median value for each year based on interpolated
% data
invs = (1/step);
max= nanmax(agemodelleddata(:,1));
min= nanmin(agemodelleddata(:,1));
n = ((max-min)*invs) - (size(agemodelleddata,1));
if n == 0;
else
    agemodelleddata = agemodelleddata(1:((max-min)*invs),:);
end
rows = size(agemodelleddata,1);

generateddata = nan((size(agemodelleddata,1)/invs),7);
generateddata(:,1) = ([max:-1:floor(agemodelleddata(end,1))+1]');

fx = [1:invs:rows]';
fy = ([invs:invs:rows]');
fxy = [fx fy];
```

```
for f=1:1:(size(fxy,1));
    generateddata(f,2) = nanmin(agemodelleddata((fxy(f,1):fxy(f,2)),3));   %d13Cmin
    generateddata(f,3) = nanmean(agemodelleddata((fxy(f,1):fxy(f,2)),3));    %d13Cmid
    generateddata(f,4) = nanmax(agemodelleddata((fxy(f,1):fxy(f,2)),3));    %d13Cmax
    generateddata(f,5) = nanmin(agemodelleddata((fxy(f,1):fxy(f,2)),5));    %d18Omin
    generateddata(f,6) = nanmean(agemodelleddata((fxy(f,1):fxy(f,2)),5));    %d18Omid
    generateddata(f,7) = nanmax(agemodelleddata((fxy(f,1):fxy(f,2)),5));    %d18Omax
end

dlmwrite('IP19_annual_stats_d13C_d18O.csv',generateddata,'precision', 6);
```

____________function_____________

```
function [otime,odata,otrough] = agemodel(itime, idepth, depth, data, step)
% AGEMODEL  function [otime,odata,otrough] = agemodel(itime, idepth, depth, data, step)
%
% This function takes data with given tie-point dates, specified at certain depths, and makes a linear timelin
% between them.  It then interpolates to a specified constant time step (i.e monthly).
%
%  [otime,odata] = agemodel(itime, idepth, depth, data, step)
%
% otime  = output time scale, evenly spaced at a given constant time step (i.e monthly)
% odata  = output data that corresponds to that time vector
% otrough = output data age modeled but no consistent time step (debugging)
% itime  = input time markers (i.e. tie points) (1 x p)\
% idepth = corresponding input depth marks for those tie points (1 x p)
% depth  = the depth that every measurement in 'data' was made (1 x m)
% data   = the data we are age modeling (1 x m)\
% step   = the constant time steps between each otime.  (i.e. 1/12) (1 x 1)

% Created by Kevin J Anchukaitis, 1st version 2006.

%% First, interpolate fully between tie points so that every data point also has a time
for place = 1:(length(idepth)-1)
  t1 = itime(place);
  t2 = itime(place+1);
  d1 = idepth(place);
  d2 = idepth(place+1);
  [d,pd1] =min(abs(depth(:,1)-d1));
  [d,pd2] =min(abs(depth(:,1)-d2));
  if place+1 == length(idepth), pd2 = length(depth); end; % deal with end points

 % Now, Linear Interpolation, simply: (percent of depth) * (time interval) + (begin time)
 for row = pd1:pd2
    otrough(row) =  (depth(row,1)-d1)/(d2-d1) * (t2-t1) + t1;
   end; % end the interpolating loop

end; % end the time/depth loop

otrough(otrough == 0) = NaN; otrough = otrough';
```

```
otrough(find(diff(otrough) ==0));

%% Now, use interpolate again to make time steps which are at a constant spacing\
if otrough(max(find(~isnan(otrough)))) > otrough(min(find(~isnan(otrough)))) % if the larger date is
later
  otime = min(itime):step:max(itime);
else
  otime = max(itime):-step:min(itime);
end

otime = otime';
odata = interp1(otrough,data,otime);

end
```

Supplemental Data References

1. Bronk Ramsey, C. Deposition models for chronological records. *Quaternary Science Reviews* **27,** 42–60 (2008).
2. Hogg, A. G. *et al.* SHCal13 Southern Hemisphere calibration, 0–50,000 cal yr BP. *Radiocarbon* (2013).
3. Hua, Q., Barbetti, M. & Rakowski, A. Z. ATMOSPHERIC RADIOCARBON FOR THE PERIOD 1950-2010. *Radiocarbon* **55,** (2013).
4. Drezner, T. Saguaro (Carnegiea gigantea, Cactaceae) age-height relationships and growth: The development of a general growth curve. *Am J Bot* **90,** 911–914 (2003).
5. Morales, M. S. *et al.* Precipitation changes in the South American Altiplano since 1300 AD reconstructed by tree-rings. *Climate of the Past* **8,** 653–666 (2012).
